# Acquisition, Maintenance and Relapse-Like Alcohol Drinking: Lessons from the UChB Rat Line

**DOI:** 10.3389/fnbeh.2017.00057

**Published:** 2017-04-04

**Authors:** Yedy Israel, Eduardo Karahanian, Fernando Ezquer, Paola Morales, Marcelo Ezquer, Mario Rivera-Meza, Mario Herrera-Marschitz, María E. Quintanilla

**Affiliations:** ^1^Molecular and Clinical Pharmacology Program, Institute of Biomedical Sciences, University of ChileSantiago, Chile; ^2^Center for Biomedical Research, Universidad Autónoma de ChileSantiago, Chile; ^3^Centro de Medicina Regenerativa, Facultad de Medicina Clínica Alemana-Universidad del DesarrolloSantiago, Chile; ^4^Department of Pharmacological and Toxicological Chemistry, Faculty of Chemical and Pharmaceutical Sciences, University of ChileSantiago, Chile

**Keywords:** ethanol, acetaldehyde, catalase, relapse, reinforcement (psychology), inflammation, stem cells, reactive oxygen species

## Abstract

This review article addresses the biological factors that influence: (i) the *acquisition* of alcohol intake; (ii) the *maintenance* of chronic alcohol intake; and (iii) *alcohol relapse-like* drinking behavior in animals bred for their high-ethanol intake. Data from several rat strains/lines strongly suggest that catalase-mediated brain oxidation of ethanol into acetaldehyde is an absolute requirement (up 80%–95%) for rats to display ethanol’s reinforcing effects and to initiate chronic ethanol intake. Acetaldehyde binds non-enzymatically to dopamine forming salsolinol, a compound that is self-administered. In UChB rats, salsolinol: (a) generates marked sensitization to the motivational effects of ethanol; and (b) strongly promotes binge-like drinking. The specificity of salsolinol actions is shown by the finding that only the R-salsolinol enantiomer but not S-salsolinol accounted for the latter effects. Inhibition of brain acetaldehyde synthesis does not influence the *maintenance* of chronic ethanol intake. However, a prolonged ethanol withdrawal partly returns the requirement for acetaldehyde synthesis/levels both on chronic ethanol intake and on alcohol relapse-like drinking. Chronic ethanol intake, involving the action of lipopolysaccharide diffusing from the gut, and likely oxygen radical generated upon catechol/salsolinol oxidation, leads to oxidative stress and neuro-inflammation, known to potentiate each other. Data show that the administration of N-acetyl cysteine (NAC) a strong antioxidant inhibits chronic ethanol *maintenance* by 60%–70%, without inhibiting its initial intake. Intra-cerebroventricular administration of mesenchymal stem cells (MSCs), known to release anti-inflammatory cytokines, to elevate superoxide dismutase levels and to reverse ethanol-induced hippocampal injury and cognitive deficits, also inhibited chronic ethanol maintenance; further, *relapse-like ethanol drinking* was inhibited up to 85% for 40 days following intracerebral stem cell administration. Thus: (i) ethanol must be metabolized intracerebrally into acetaldehyde, and further into salsolinol, which appear responsible for promoting the *acquisition* of the early reinforcing effects of ethanol; (ii) acetaldehyde is not responsible for the *maintenance* of chronic ethanol intake, while other mechanisms are indicated; (iii) the systemic administration of NAC, a strong antioxidant markedly inhibits the *maintenance* of chronic ethanol intake; and (iv) the intra-cerebroventricular administration of anti-inflammatory and antioxidant MSCs inhibit both the *maintenance* of chronic ethanol intake and *relapse-like drinking*.

## Introduction

This review addresses the biological factors that influence: (i) the *acquisition* of alcohol intake; (ii) the *maintenance* of chronic alcohol intake; and (iii) *alcohol relapse-like* drinking behavior in animals bred for their high-ethanol intake.

Two animal lines derived from the Wistar strain generated 60 years ago (see Mardones and Segovia-Riquelme, 1983; Quintanilla et al., 2006) have been kept for over 90 generations by selective and genetic breeding. These are: an Abstainer rat (UChA) line and a high-ethanol drinker line referred to as Bibulous rat (UChB). The mechanisms leading to alcohol rejection in the virtually abstainer UChA line are mainly related to polymorphisms in nuclear and mitochondrial genes that lead to a slow metabolism of acetaldehyde and to high blood acetaldehyde levels. These UChA studies have been previously described (Sapag et al., 2003; Quintanilla et al., 2005, 2006; Israel et al., 2013) and thus not covered in the present review. Studies conducted in UChB rats are indicated in the text.

### Alcohol-Use Disorders: Genetic Aspects

Since alcoholism is 40%–60% genetically determined (Heath et al., 1997; Li, 2000), animals bred to consume high amounts of alcohol while on a constant environment might provide an answer for an elusive single “alcoholism gene” or the lack thereof. It took years for science to conclude that there is no single gene that could promote a high ethanol intake. Such a view could already be derived from crosses between inbred mice with markedly different ethanol intakes: the second generation (F_2_) of crosses between high-intake (C57BL) and low intake animals (DBA) results in animals presenting the complete alcohol intake phenotype spectrum spanning their original strains (Phillips et al., 1994).

While in human and animals, analyses of hundreds of genes and genome-wide studies indicate that several polymorphisms or chromosomal markers correlate with alcohol intake and/or alcohol use disorders, these polymorphisms/markers have only minor effects in predicting alcohol-use disorders, compared to the marked effect of the polymorphisms of genes coding for alcohol and acetaldehyde metabolizing enzymes. The reader is referred to a recent review in this area (Tawa et al., 2016).

## Acquisition of Ethanol Intake

### The Reinforcing Effect of Ethanol-Derived Brain Acetaldehyde

A number of studies in laboratories in Spain, Chile and Italy, using Sprague-Dawley, UChB or Wistar rats, respectively (Tampier and Mardones, 1979; Aragon and Amit, 1992; Peana et al., 2008) have indicated that acetaldehyde generated in the brain by the action of catalase mediates the ethanol reinforcing mechanism. Acetaldehyde generated by the hepatic metabolism of ethanol and present in blood at levels within 10–50 μM, does not cross the blood brain barrier (Eriksson et al., 1977; Lindros and Hillbom, 1979; Petersen and Tabakoff, 1979; Stowell et al., 1980). However, large doses of exogenous acetaldehyde are able to overcome the blood brain barrier limitation. It has been shown that a single intraperitoneal injection of 50 mg acetaldehyde/kg, resulting in blood levels of 350–400 μM acetaldehyde, doubles voluntary ethanol intake in UChB rats (Quintanilla and Tampier, 2003). Thus, a large dose of acetaldehyde sensitizes ethanol reinforcement in UChB rats. This sensitizing effect may be mediated by brain-generated salsolinol formed by the condensation of acetaldehyde and dopamine (*vide infra*).

Operant self-administration studies have shown that rats bred as alcohol high-drinkers (Indiana University, P and HAD rats) will bar-press to self-administer both ethanol and acetaldehyde into the ventral tegmental area (VTA). Noteworthy, the levels of acetaldehyde required for self-administration into the VTA are three orders of magnitude lower for acetaldehyde, in the range of 10 μM than those for ethanol, which are in the range of 10–20 mM (Rodd et al., 2005). These studies indicate that as a reinforcing agent acetaldehyde is more potent than ethanol.

**Figure 1:**
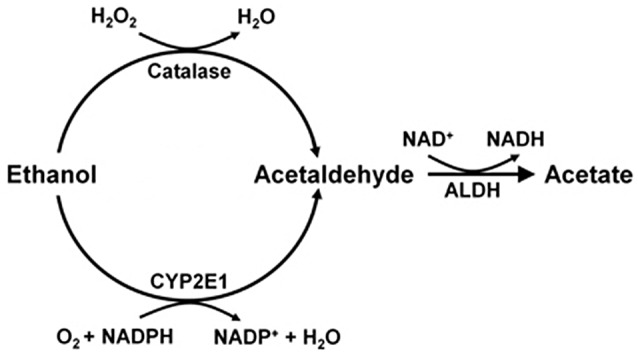
**Ethanol metabolism into acetaldehyde and acetate in the brain (Scheme from data of Zimatkin et al., 2006)**.

Studies on the mechanisms that generate brain acetaldehyde in Wistar rats show that catalase is responsible for 70% of the brain oxidation of ethanol into acetaldehyde (Zimatkin et al., 2006). Acetaldehyde is rapidly converted into acetate, likely via a low Km aldehyde dehydrogenase (ALDH). In the presence of ethanol, acetate levels in brain homogenates are 7-fold greater than those of acetaldehyde (Zimatkin et al., 2006). Studies by Zimatkin et al. (2006) also suggest that 15% of brain acetaldehyde is generated from CYP2E1 (Figure 1).

**Figure 2:**
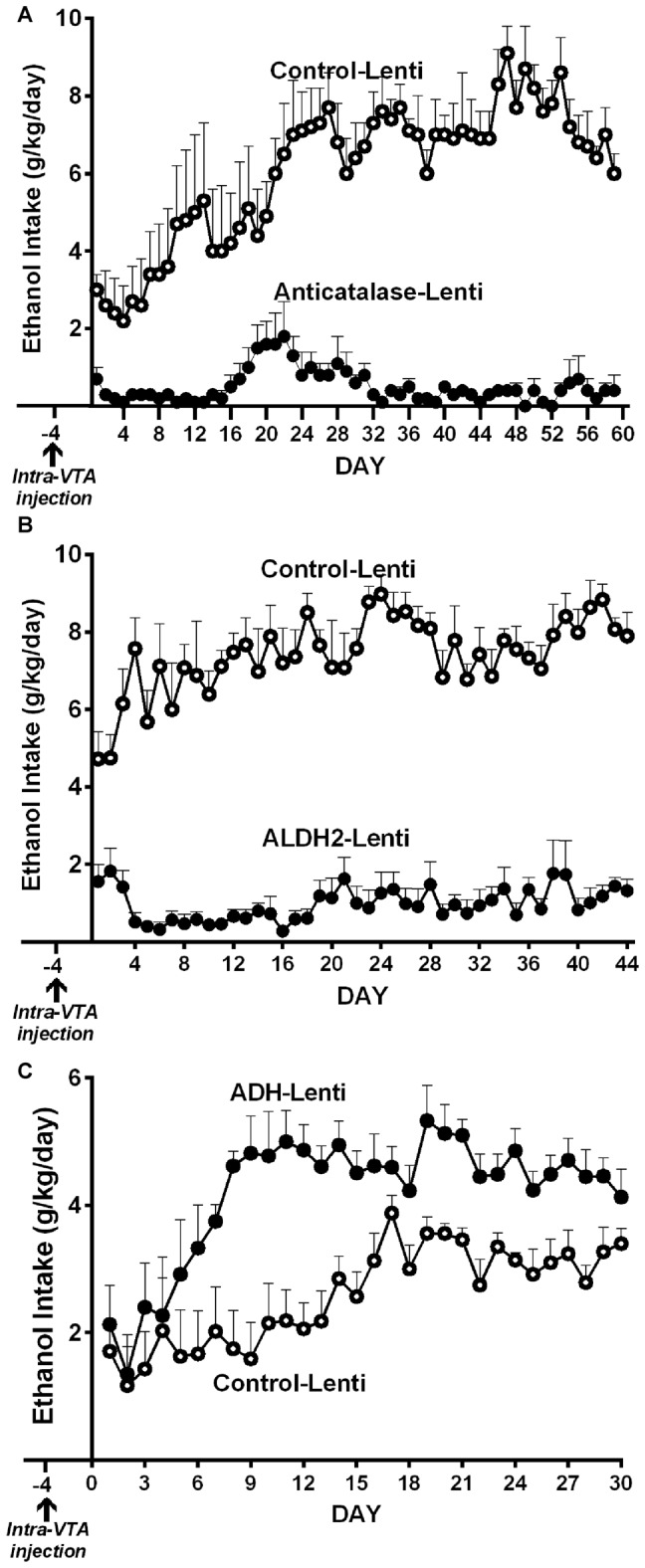
**Anticatalase-Lentiviral vector (A)** or aldehyde dehydrogenase-2 (ALDH2)-coding Lentiviral vector **(B)** injection into the ventral tegmental area (VTA) of naïve UChB rats markedly reduces voluntary ethanol intake. A liver alcohol dehydrogenase (ADH)-coding Lenti vector injection **(C)** significantly increases ethanol intake in ethanol naïve rats. Arrow indicates the time of administration of either control-lentiviral vector, anticatalase- **(A)**, ALDH2-lentiviral vector **(B)** or ADH-lentiviral vector (*n* = 5 rats per group). UChB rats significantly (*p* < 0.001) reduced their alcohol intake (10% v/v) when injected into the VTA with a lentiviral vector coding for: (i) a shRNA against catalase (anticatalase-Lenti) **(A)**; or (ii) ALDH2-coding Lentiviral vector **(B)**, compared to animals injected with an empty lentiviral vector (control-Lenti). Rats significantly (*p* < 0.01) increased their alcohol l (5% v/v) intake when injected with a lentiviral vector coding liver ADH **(C)** (Panel** A,C** were adapted from Karahanian et al., 2011 and Panel** B** was adapted from Karahanian et al., 2015).

Two types of studies conducted in UChB rats strongly suggest that the generation of acetaldehyde in the VTA is *an absolute requirement for the acquisition* of alcohol reinforcement:

(a) *Genetic inhibition of catalase synthesis*. Figure 2A shows that the intra VTA administration of a lentiviral vector coding for an anticatalase shRNA blocked ethanol intake by 95%. It is noted that ethanol administration to Sprague-Dawley and UChB rats significantly increases dopamine release in nucleus accumbens (Imperato and Di Chiara, 1986; Di Chiara and Imperato, 1988; Quintanilla et al., 2007; Bustamante et al., 2008), while blocking the synthesis of catalase by the injection of the lentiviral vector coding for an shRNA anticatalase abolished the increases in dopamine release induced by ethanol administration. Such an effect, studied in the UChB rat, was specific for ethanol, since dopamine release induced by amphetamine or KCl depolarization was not changed by the intra VTA administration of the shRNA anticatalase coding lentiviral vector (Karahanian et al., 2011)
(b) *Transducing a gene encoding an enzyme that degrades acetaldehyde*. The administration to naïve UChB rats of a lentiviral vector coding for the wildtype high affinity Aldh2, aimed at increasing the VTA ability to degrade acetaldehyde resulted in an 85% inhibition of ethanol (10% v/v) intake (Karahanian et al., 2015; Figure 2B).

In addition to the above, transducing into the VTA a gene coding for liver alcohol dehydrogenase (ADH), an enzyme that generates acetaldehyde, *increased* 2–4 fold the reinforcing effect of 5% ethanol (Karahanian et al., 2011; Figure 2C).

The above studies would preclude other brain systems in the *acquisition* (development) of ethanol reinforcement in rats.

### Systemic Acetaldehyde Can be Both Aversive and Reinforcing

Systemic acetaldehyde generated endogenously at levels that do not cross the endothelial cell layer of the blood brain barrier is aversive (e.g the acetaldehyde protection against alcoholism of East Asians carrying the ALDH2*2 genotype). However high systemic concentrations of acetaldehyde prior reached when acetaldehyde itself is orally consumed or it is administered intraperitoneally which can cross the blood brain barrier and are reinforcing, as shown by Peana et al. (2010, 2011) in a nose-poking for oral acetaldehyde in an operant model in Wistar rats. Although blood acetaldehyde levels were not reported, these studies further support the reinforcing-motivational role of brain acetaldehyde and are in line with studies of Diana and associates (Foddai et al., 2004) who postulated a preferential reinforcing effect of systemic acetaldehyde over its aversive effects. In UChB rats, Quintanilla and Tampier (2003) showed that the injection of a large dose of acetaldehyde results in conditioned place preference (CPP). Early studies by Brown et al. (1980) demonstrated that infusion of acetaldehyde directly into the left lateral ventricle of the brain of Wistar rats leads to increases in ethanol intake. In this case the peripheral aversive effects are expected to be minimal.

These findings, together with microdialysis studies (Melis et al., 2007; Deehan et al., 2013a,b) show that local administration of acetaldehyde into the posterior VTA leads to increases in dopamine release in nucleus accumbens further suggesting that the reinforcing effect of acetaldehyde is mediated by activation of dopaminergic neurons. Thus, the effect of acetaldehyde on dopamine release mimics the effects of many drugs of abuse (Di Chiara and Imperato, 1988).

Overall, literature studies support the view that brain acetaldehyde is reinforcing. The possibility that acetaldehyde may be converted into another reinforcing substance is subsequently discussed.

### The Reinforcing Effect of Salsolinol: An Acetaldehyde-Derived Product

**Figure 3:**
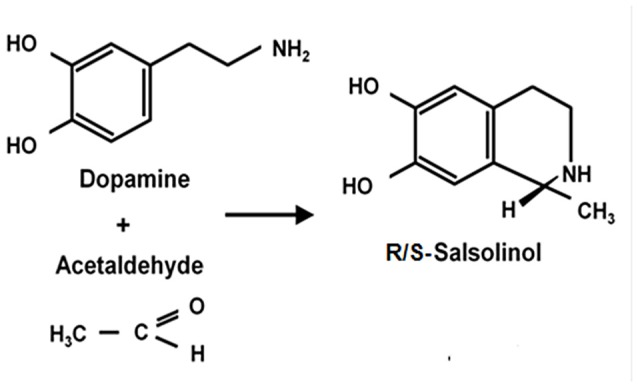
**Condensation of dopamine and acetaldehyde yielding salsolinol**. Schematic representation of the spontaneous condensation of acetaldehyde with dopamine, yielding salsolinol (adapted from King et al., 1974; Bates et al., 1986).

Ethanol-derived acetaldehyde condenses non-enzymatically with brain dopamine to generate racemic (*R/S*)-salsolinol (*R/S*-SAL; Figure 3). Rodd et al. (2008) and Deehan et al. (2013b) have shown that (*R/S*)-SAL at concentrations of 0.03–0.3 μM is self-administered intra VTA by Wistar rats. These concentrations of (*R/S*)-SAL are one to two orders of magnitude lower than the concentrations required for acetaldehyde self-administration in the same brain area.

Microinjections of (*R/S*)-SAL into the VTA of Wistar rats also result in an increased release of dopamine in the nucleus accumbens (Deehan et al., 2013a,b). Rommelspacher et al. (1995) showed that SAL was increased in the blood of alcoholics. Animal studies have shown that chronic ethanol administration to Sprague-Dawley and to high alcohol drinker (HAD) rats results in a significant increase of SAL levels in dopamine-rich areas of the brain (Sjöquist et al., 1982; Matsubara et al., 1987; Rojkovicova et al., 2008).

Several questions arise in relation to the action of SAL: (i) is endogenous dopamine required to generate SAL? (ii) does the chronic administration of (R/S)-SAL generate a sensitized state similar to that generated by chronic ethanol intake, which augments ethanol reinforcement? (iii) does (R/S)-SAL administration to naïve rats induce ethanol motivational effects? (iv) is there an enantiomer specificity distinguishing the effects of R-salsolinol vs. S-salsolinol; and (v) does (R/S)-SAL administration result in locomotor sensitization, as it happens after chronic ethanol administration?

**Figure 4:**
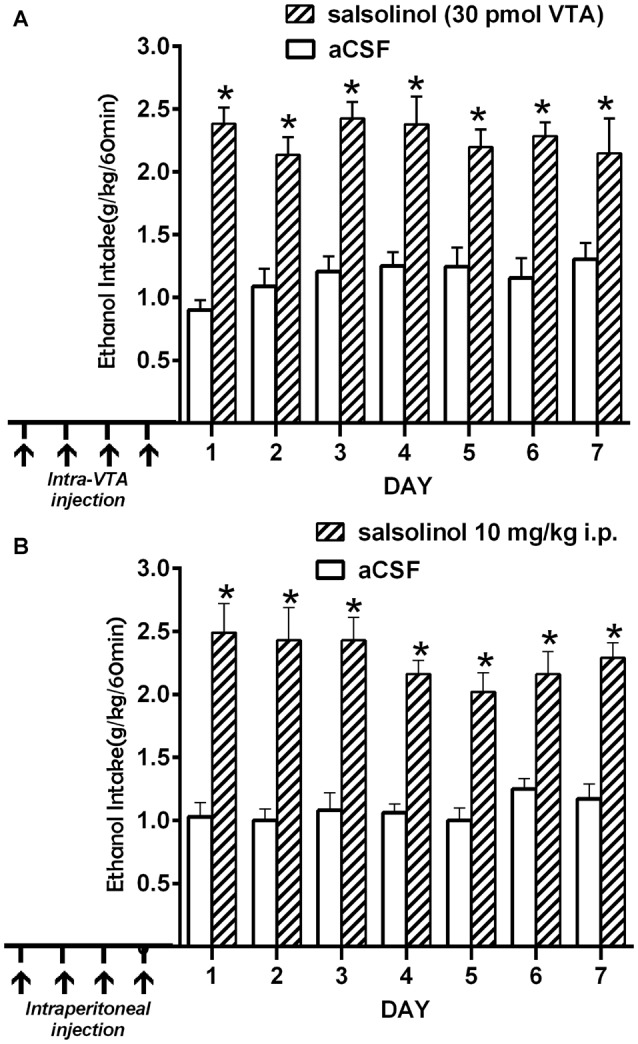
**Prior salsolinol administration increases voluntary ethanol intake in UChB rats. (A)** UChB rats (*n* = 5) pretreated with salsolinol into the VTA (30.0 pmol/0.2 μl; days 1, 3, 8, 12; *arrows*) increased ethanol intake (60 min/day) during seven consecutive days (*stippled bars*), vs. rats pretreated with artificial cerebrospinal fluid (aCSF; *n* = 5) into the VTA (0.2 μl; days 1, 3, 8, 12; arrows; *white bars*). **(B)** UChB rats (*n* = 5) pretreated with systemic salsolinol (10 mg/kg, i.p.; days 1, 3, 8, 12; *arrows*) increased ethanol intake (60 min/day) during seven consecutive days (*striped bars*), vs. UChB rats (*n* = 5) pretreated with saline (7 ml/kg, i.p.; days 1, 3, 8, 12; arrows; *white bars*) (Data from Quintanilla et al., 2014). Symbol * means significant difference from aCSF control rats *p* < 0.001 (Two way ANOVA).

**Figure 5:**
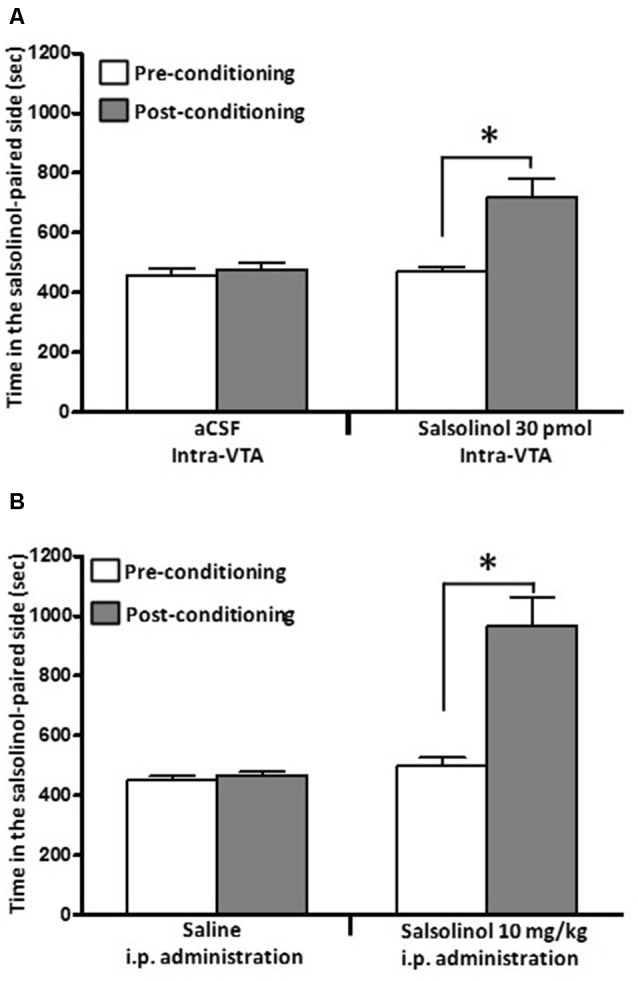
**Salsolinol, injected intracerebrally or systemically, produced conditioned place preference (CPP) in UChB rats**. Salsolinol or vehicle was administered either **(A)** intra-VTA (30 pmol/left VTA) or **(B)** intraperitoneally (10 mg/kg, i.p.) in UChB rats (*n* = 28). Data are means ± SEM and represent the time spent in the salsolinol-paired compartment (seconds; means ± SEM, *n* = 7 for each group; ordinate) during the pre- (white columns) and postconditioning (gray columns) phases. Asterisk represents significant difference in time spent by the salsolinol group on the salsolinol-paired side in the postconditioning phase vs. the preconditioning phase and vs. the vehicle group: *p < 0.001 (two-way ANOVA) (Data from Quintanilla et al., 2014).

**Figure 6:**
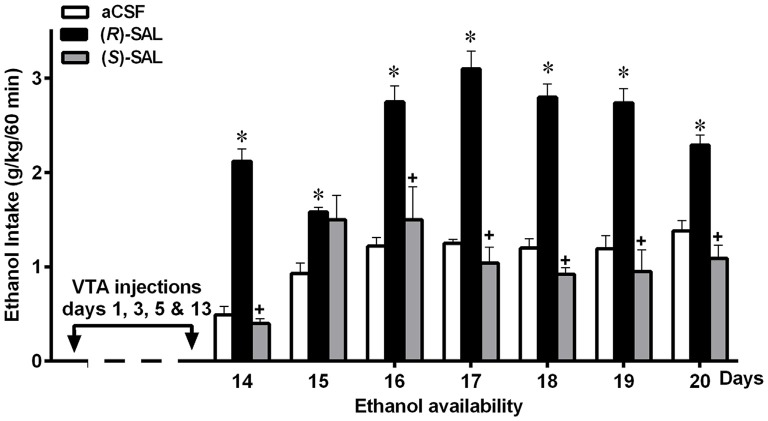
**Prior repeated administration of (*R*)-SAL, but not of (*S*)-SAL, increases voluntary ethanol intake in ethanol-naïve UChB rats**. UChB rats (*n* = 5 rats per group) were pretreated intra-VTA with (*R*)-SAL (black columns), (*S*)-SAL (gray columns) or control aCSF (white columns) on days 1, 3, 5 and 13 (*arrows*); then (from day 14) animals were exposed to ethanol for 1 h/day. Asterisk symbol (*P < 0.05) indicates that the ethanol intake is significantly higher than that of the control (aCSF) group of the same day. Single plus (+ cross-like) sign indicates that the ethanol intake of (S)-SAL treated animals is significantly different lower (*p* < 0.001) than that of the (R)-SAL group on the same day (Data from Quintanilla et al., 2016a).

As will be discussed below; the answer to all these questions is “yes”:

(i) *In vitro* studies by Melis et al. (2015) showed that inhibition of dopamine synthesis by α-methyl-p-tyrosine, a tyrosine hydroxylase inhibitor, fully abolishes the ability of ethanol and acetaldehyde to activate VTA dopaminergic neurons, an effect that was specific for ethanol and acetaldehyde (as SAL precursors) but was not seen for pre-formed SAL.
(ii) Intra-cerebroventricular (Figure 4A) or systemic administration of (*R/S)*-SAL (Figure 4B) to ethanol naïve UChB rats induces major increases in voluntary ethanol intake (Quintanilla et al., 2014). This effect was also observed by Myers and Melchior (1977) in Sprague-Dawley rats. Noteworthy, the high ethanol intakes are of the same order as those ingested by rats that had consumed ethanol for several weeks and were exposed to the ethanol deprivation condition followed by ethanol re-access (*vide infra*).
(iii) Studies by Quintanilla et al. (2016a), in UChB rats showed that the intra-cerebroventricular or systemic administration of (*R/S)*-SAL increased the motivational effects of ethanol as shown by the place preference technique (Figure 5), in line with studies by Matsuzawa et al. (2000) in Sprague-Dawley rats and by Hipólito et al. (2011) in Wistar rats.
(iv) Intracerebral administration studies in UChB rats showed that the ethanol motivational and intake sensitization effects of (*R/S)*-SAL is also seen with R-SAL while the S-SAL enantiomer is inactive (Figure 6; Quintanilla et al., 2016a). Although the pharmacological mechanisms responsible for the action of *(R/S*)-SAL remain unclear, the specific effect of the R-enantiomer in inducing the motivational effects of ethanol, suggests that *in vivo* the chirality of the C-1 center of *(R/S*)-SAL plays an important role in changing its affinity for transporters or receptors associated with ethanol intake.
(v) The administration of (R/S)-SAL to Wistar or UChB rats induced a sensitization to its locomotor effects (Hipólito et al., 2010; Quintanilla et al., 2014).

In addition to the above, the findings that microinjections of salsolinol into the posterior VTA increase dopamine release in nucleus accumbens (Hipólito et al., 2011; Deehan et al., 2013a) suggest that the reinforcing effect of salsolinol is mediated by the activation of dopaminergic neurons.

Overall, the above studies suggest that brain SAL mediates the effect of ethanol-derived acetaldehyde to motivate the acquisition of ethanol consumption. Further, these studies are also in line with the work of Rodd and associates (Rodd et al., 2008; Deehan et al., 2013b) who showed that rats will self-administer (R/S)-SAL into the posterior VTA at concentrations that are below those required for acetaldehyde self-administration.

## Maintenance of Chronic Ethanol Intake

### Ethanol-Derived Acetaldehyde Is no Longer Required to Maintain Chronic Alcohol Intake

**Figure 7:**
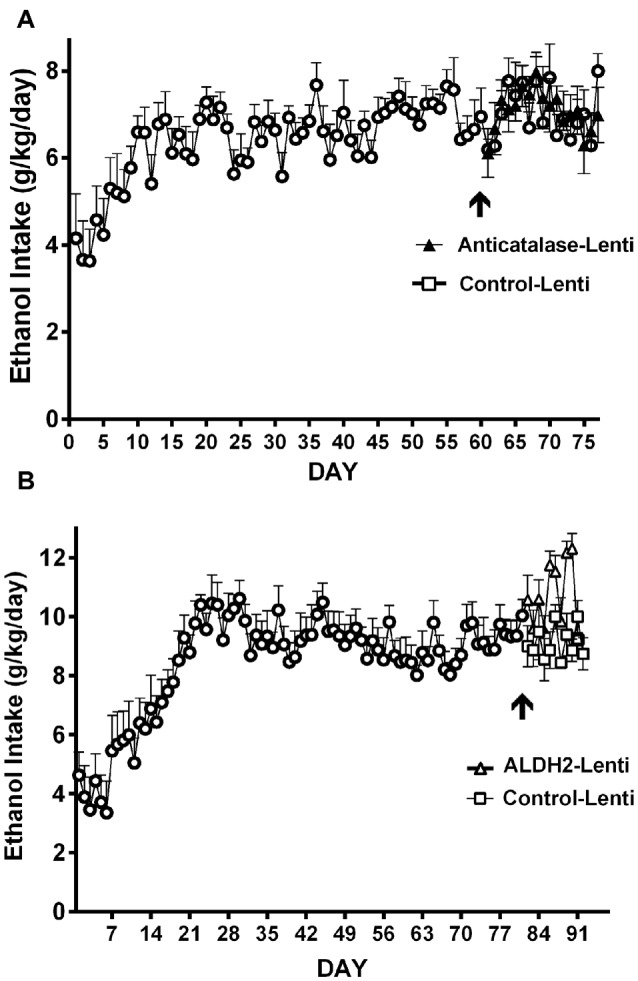
**Injection of an shRNA anti-catalase (A)** or ALDH2 coding vector **(B)** into the VTA did not reduce alcohol (10%) intake in UChB rats that had chronically ingested ethanol. Arrow indicates the time of administration in **(A)** control-lentiviral vector (-□-) or anticatalase-lentiviral vector (-▲-) and in **(B)** the control-lentiviral vector (-□-) or the ALDH2-coding vector (-△-). The number of animals was 8 or 10 rats/group. Neither the anticatalase, the ALDH2-coding vector nor the control vector modified the voluntary ethanol intake of the animals that had already been under free choice ethanol consumption for 60 days (Panel **A** was adapted from Quintanilla et al., 2012 and Panel **B** was adapted from Karahanian et al., 2015).

Studies by Quintanilla et al. (2012) and Karahanian et al. (2015) have shown that *after* the UChB rats have reached a steady state of chronic ethanol intake, the administration into the VTA of either a lentiviral vector coding for an anti-catalase shRNA (Figure 7A) or coding for the high affinity Aldh2 do not influence voluntary ethanol intake (Figure 7B). It is noted that the unabated ethanol intake seen in these studies after the transduction of genes aimed at lowering acetaldehyde levels is not due to negative reinforcement since addition of quinine (bitter taste) to the ethanol solutions fully inhibits ethanol intake (Quintanilla et al., 2012).

The failure of the anticatalase or ALDH2 coding lentiviral vectors to reduce ethanol intake in rats that had consumed alcohol for 2 months suggests that following chronic alcohol consumption other signaling pathways might be recruited. Chronic consumption of drugs of abuse induces changes at the molecular, cellular and neurocircuitry levels that mediate the transition from occasional, controlled substance use to loss of control in drug intake and chronic addiction (Koob and Le Moal, 2008). Although chronic drug consumption induces changes in several neurotransmitter systems, including dopamine, GABA, cannabinoids and opioid systems (see Fattore and Diana, 2016), without implying a lesser role for other neurotransmitter systems, we mainly focus our review on glutamate transmission since: (i) many addictive drugs lead to an increased glutamatergic signaling (Wolf et al., 2003; Koob and Volkow, 2010); which (ii) drive the enhanced motivation to obtain several drugs, including cocaine (Kalivas and McFarland, 2003; Pickens et al., 2011) and, now also ethanol (Sari et al., 2013; Das et al., 2015); and (iii) can be modified by the administration of drugs that normalize the glutamate homeostasis (*vide infra*).

### The Hyperglutamatergic Hypothesis

Mechanisms that lead subjects to maintain drug intake involve: (a) learned cues (see Volkow et al., 2002; Hyman et al., 2006; Berridge et al., 2009); for ethanol likely its odor (see Bragulat et al., 2008); and (b) an increased glutamatergic tone (see Reissner and Kalivas, 2010). An increased glutamatergic tone has been shown in Sprague-Dawley rats to be associated with the *maintenance* of chronic ethanol intake, as well as with the relapse of several drugs of abuse (Weiland et al., 2015). Alcohol preferring rats (P strain) on a chronic ethanol intake schedule show marked increases in extracellular glutamate in nucleus accumbens (Ding et al., 2012), resulting from a reduction in the levels of the Na^+^-glutamate (exchange) transporter (GLT1) in astrocytes of tripartite glutamatergic synapses. Sari et al. (2013) studied the *maintenance* of chronic ethanol intake in P rats. In these studies, the administration of ceftriaxone, a drug that increases the levels of GLT1, resulted in a 60%–70% reduction in chronic ethanol intake. Das et al. (2015) confirmed an elevation of extracellular glutamate in nucleus accumbens of  P rats exposed to ethanol chronically, and showed that ceftriaxone markedly inhibited chronic ethanol intake.

Studies in Sprague-Dawley rats show that extracellular glutamate levels are regulated not only by the astrocyte Na+ gradient-dependent GLT1 transporter but also by the astrocyte exchange of cystine for glutamate via the cystine/glutamate exchanger (Herrera-Marschitz et al., 1996; Baker et al., 2003). Scofield and Kalivas (2014) demonstrated that a number of drugs of abuse reduce the levels of the GLT1 transporter and increase the extracellular levels of glutamate. In rodents, operant cocaine self-administration reduces both the nucleus accumbens cystine-glutamate exchange and glutamate transport by the GLT-1 transporter. Most importantly, administration of the antioxidant drug N-acetyl cysteine (NAC) normalizes these two processes. Reissner et al. (2015) concluded that restoring GLT-1, not the cystine-glutamate exchange, is the key mechanism whereby daily NAC reduces the hyperglutamatergic state.

### The Administration of N-Acetyl Cysteine in Drug Dependence and Motivation

A number of studies have shown that NAC reduces relapse (or reduces CPP) of many addictive drugs including: (i) cocaine (Madayag et al., 2007; Moussawi et al., 2009; Reichel et al., 2011; Kupchik et al., 2012; Reissner et al., 2015); (ii) nicotine (Ramirez-Niño et al., 2013; Bowers et al., 2016; Moro et al., 2016); (iii) heroin (Zhou and Kalivas, 2008); and now (iv) ethanol (Quintanilla et al., 2016b).

Most of the above studies were conducted in rodents, while only one clinical study has reported the effect of NAC on a drug use disorder (LaRowe et al., 2013). These investigators indicated that their studies failed to demonstrate that NAC reduced cocaine use in cocaine-dependent individual actively self-administering the drug. However, they also report that NAC prevented the return to cocaine use (relapse) in individuals who had already achieved abstinence from cocaine. In the latter, NAC administration reduced cocaine relapse by 90% (LaRowe et al., 2013; Figure 2). Despite the promising studies showing an inhibition of cocaine relapse in animals, these studies in abstinent cocaine users will require confirmation before their clinical application.

A possible explanation for the effect of NAC in preventing cocaine relapse only in detoxified patients may relate to the dual effect of cocaine in generating oxygen radicals (ROS) in the brain: (i) cocaine inhibits both the dopamine transporter and the norepinephrine transporter (Dohi et al., 2002), thus increasing the exposure of both extracellular dopamine and norepinephrine to a physiological pH, which leads to the autooxidation of catecholamines, generating one-electron oxidant semi-quinones; and (ii) both neurotransmitters are deaminated by monoamine oxidases generating hydrogen peroxide (Kopin, 1994). Thus, NAC is expected to be considerably less active when cocaine continues to be self-administered, while most active in a condition where only the *remaining* cocaine-induced ROS self-potentiating effects promote drug relapse. Amphetamine-like drugs have similar properties as cocaine in generation of ROS as amphetamines release not only dopamine but also norepinephrine (Rothman et al., 2001). McClure et al. (2014) suggest that NAC may prove to be an ideal relapse prevention aid when given after periods of abstinence or when combined with other forms of pharmacological and/or behavioral treatments to promote abstinence.

Despite the above, NAC markedly inhibited the chronic intake of ethanol of rats (*vide infra*). Animals were chronically self-administering alcohol; thus not in an abstinent condition (Quintanilla et al., 2016b), suggesting that the ROS/neuroinflammation generated by chronic ethanol intake is less intense than that generated by drugs that increase the extracellular levels of both dopamine and norepinephrine. We are not aware of clinical studies aimed at testing the effect of NAC as a treatment of alcoholism, whether prior or after abstinence. However, a recent clinical study showed that when successful, NAC treatment of marihuana users also reduced their alcohol use (Squeglia et al., 2016).

**Figure 8:**
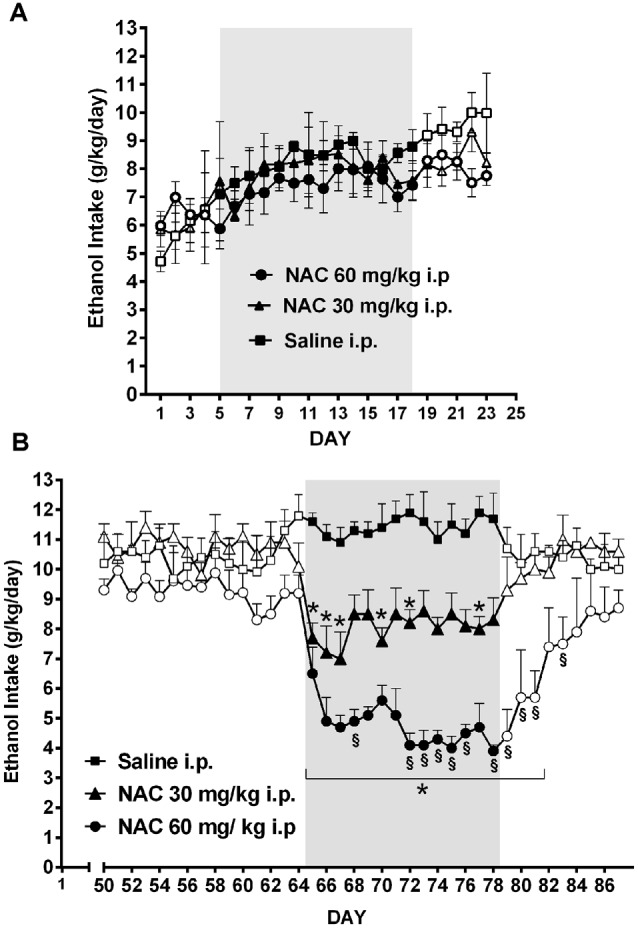
**Chronic N-acetyl cysteine (NAC) administration (30 or 60 mg/kg, i.p.) administered during the acquisition of alcohol consumption did not reduce ethanol intake (A)**, but given during the chronic ethanol maintenance phase markedly reduced ethanol intake **(B)** in UChB rats. Three groups of rats (*n* = 5 rats per group) under continuous access to 10% ethanol and water were treated with either saline (black square), 30 mg/kg NAC i.p. (black triangles), or 60 mg/kg N AC i.p (black circles) from day 5–18 (acquisition phase) of ethanol intake **(A)**. Different groups of rats under continuous access to 10% ethanol and water rats (*n* = 5 rats per group) were treated with either saline (black square), 30 mg/kg NAC (black triangles), or 60 mg/kg NAC (black circles) from day 65 to day 78 (maintenance phase) of ethanol intake **(B)**. Data are means ± SEM of daily ethanol intake. Asterisk symbol *p < 0.05, indicates that the ethanol intake is significantly lower than that of the saline control group of the same day. (§) Symbol *p* < 0.001, indicates that the ethanol intake of NAC 60 mg/kg group is lower than that of NAC 30 mg/kg group (Data from Quintanilla et al., 2016b).

As shown by Quintanilla et al. (2016b) the daily administration of NAC, although not inhibiting the *acquisition* of chronic ethanol intake (Figure 8A), was a strong inhibitor (70%–75% reduction) of ethanol intake *maintenance* (Figure 8B). These results are in line with the findings of Doyle et al. (2014) indicating that glutamatergic signaling in the nucleus accumbens of Sprague-Dawley rats, although not essential for modifying initial cocaine use in non-addicted stages, becomes critical for post withdrawal relapse after the addiction has developed.

### Oxidative Stress and Neuroinflammation: A General Role in the Addiction Process

Several studies indicate that oxidative stress is a relevant mechanism contributing to neural cytotoxicity and behavioral changes associated with drug addiction (see Cunha-Oliveira et al., 2013). Oxidative stress in the nervous system has been found upon *in vivo* exposure to amphetamine or amphetamine derivatives (Frey et al., 2006; Jung et al., 2010) and heroin (Qiusheng et al., 2005; Xu et al., 2006). Withdrawal from cocaine or heroin also induces oxidative stress in rodent’s brain (Cemek et al., 2011; Pomierny-Chamiolo et al., 2013). The effect NAC in both normalizing glutamate levels and reducing drug relapse (Reissner et al., 2015) likely results from its high antioxidant activity. N-acetylcysteine is also used to treat an acetaminophen overdose due to its high antioxidant activity, being a precursor of cysteine and glutathione (Lucyk et al., 2016).

A number of studies in Wistar and Sprague-Dawley rats have shown that chronic ethanol administration leads *to both oxidative stress* and *neuroinflammation* (reviewed by Crews et al., 2015; Crews and Vetreno, 2016). Noteworthy, oxidative stress and neuroinflammation potentiate each other via the oxidation of IκB with activation of NFκB and the generation of inflammatory cytokines; the latter in turn generate oxygen radicals via mitochondrial uncoupling (Kastl et al., 2014). Montesinos et al. (2016) reviewed the direct relationship between neuroinflammation and brain injury. In alcoholics, a marked hippocampal cell loss and injury has been shown (Sullivan et al., 1995). Long Evans rats that consume alcohol for several months in nutritionally adequate liquid diets also display marked hippocampal damage (Walker et al., 1980). Studies in C57/BL mice have shown that chronic alcohol intake increases brain TLR4 and NF-κB, both involved in the generation of inflammatory cytokines (Alfonso-Loeches et al., 2010).

In a most relevant study *causally linking neuroinflammation to* an increased ethanol intake, Blednov et al. (2011) showed long-lasting increases in ethanol intake in C57/BL mice following the administration of a single dose of bacterial lipopolysaccharide, a well know neuroinflammatory agent, thus increasing the reinforcing effect of ethanol. Ethanol intake, via gut-generated acetaldehyde, induces the entrance of intestinal lipopolysaccharide into the blood (Ferrier et al., 2006), which via TNF-α generates neuroinflammation (see Crews et al., 2015). Additionally, in the brain itself a pro-oxidant and pro-inflammatory agent generated from ethanol is salsolinol; an oxygen radical-generating agent when oxidized into semi-quinones by metals ions present in biological systems (Jung and Surh, 2001). Thus, oxygen radicals and lipopolysaccharide potentiate each other in generating a neuroinflammation.

An additional link between neuroinflammation and an increased glutamatergic signal has been recently reported (David et al., 2016). The authors demonstrated a significant reduction in the primary astrocytic glutamate transporter, GLT-1 and increases in extracellular glutamate levels induced by neuroinflammation following an infection due to toxoplasma administration to mice. Thus, these studies further support the sequence oxidative stress/neuroinflammation—low glutamate transporter—hyperglutamatergic state.

Overall, the studies reviewed indicate that different mechanisms are responsible for the *acquisition of ethanol intake* and for its chronic *maintenance*. A number of studies strongly support the view that chronic ethanol intake is maintained by mechanisms known to increase the extracellular levels of brain glutamate, likely in nucleus accumbens. The inhibition of chronic ethanol intake by NAC, a strong antioxidant, further suggests that the reactive oxygen species/neuroinflammation system plays a role in chronic ethanol *maintenance*. The reactive oxygen radical species and inflammatory cytokines (e.g., TNF-alpha) are known to potentiate each other.

## Relapse-Like Alcohol Intake

### Following Ethanol Deprivation, Ethanol Intake Upon Re-Access Is Again Dependent on Brain Acetaldehyde

**Figure 9:**
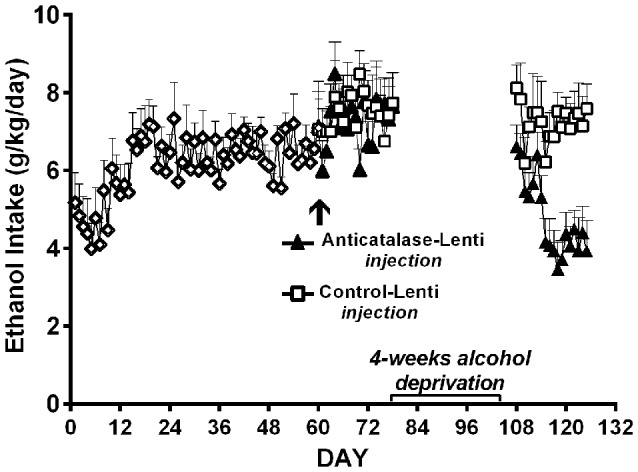
**A period of alcohol deprivation is required to reduce voluntary alcohol intake following the anticatalase-lentiviral vector administration into the VTA in UChB rats**. UChB rats (*n* = 10 rats per group) allowed to access 10% ethanol and water on a 24-h basis for 2 months did not change their voluntary alcohol intake when these were subsequently injected into the VTA with a single dose of a lentiviral vector coding for an shRNA against catalase (anticatalase-Lenti), but significantly (ANOVA; *F*_(1,33)_ = 111.54, *p* < 0.001) reduced (50%) their alcohol intake following 4 weeks of alcohol deprivation when compared to the ethanol intake of animals (*n* = 10) that received a control lentiviral vector (control-Lenti). After the alcohol deprivation period, the animals were returned to a free access of 10% ethanol and water on a 24-h basis (Data from Quintanilla et al., 2012).

In UChB rats ingesting ethanol chronically for 2-months, a 4-week ethanol deprivation leads to a partial recovery of the inhibitory effect of the anti-catalase vector on ethanol intake (Figure 9; Quintanilla et al., 2012). The fact that the effect on 24-h ethanol intake is not seen immediately after ethanol re-access may relate to the marked intake in the first few hours following the alcohol deprivation and re-access, in line with studies of Hölter and Spanagel (1999); Rodd et al. (2009) and Vengeliene et al. (2014) for Wistar and HAD rats.

The relapse-like drinking also known as the “alcohol deprivation effect” (ADE) is a condition in which animals subjected to chronic ethanol intake followed by a long deprivation, consume intoxicating amounts of ethanol in as little as 60-min upon ethanol re-access. The ADE paradigm in animals has good predictive value in representing relapse-like drinking in humans as it is inhibited by three medications used clinically to reduce ethanol intake, namely: naltrexone, nalmefene and acamprosate-Ca (Spanagel and Zieglgänsberger, 1997; Orrico et al., 2014; Spanagel et al., 2014), indicating that several neurotransmitter systems- including a hyperglutamatergic tone and importantly the opiate system also mediate ADE. As will be discussed below, acetaldehyde also plays a role in relapse-like ADE drinking.

**Figure 10:**
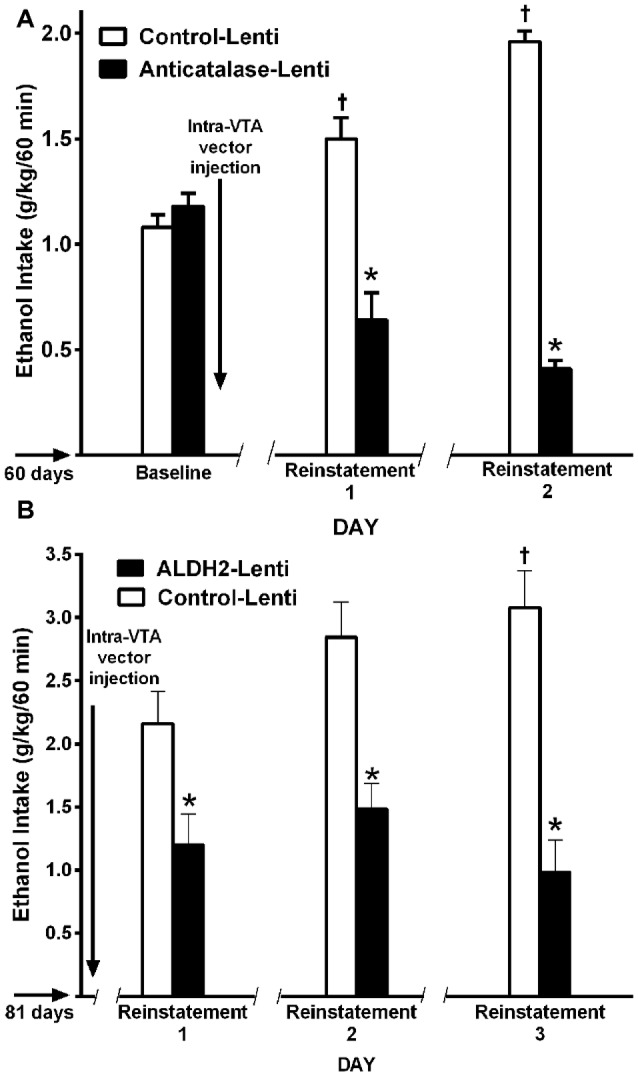
**A single VTA area injection of a shRNA anticatalase-coding lentiviral vector (A)** or an ALDH2-coding lentiviral vector **(B)** inhibit first-hour ethanol (EtOH) relapse-like ethanol intake after the first and second and third deprivation periods. Baseline data correspond to the average of EtOH intake restricted to only 1 h a day, for 7 days immediately prior to alcohol deprivation before intra-VTA injections **(A,B)** by UChB rats. Control-Lenti or Anticatalase-Lentiviral vector (*n* = 5 rats per group) were injected during the first day of deprivation **(A)**. Control-Lenti or ALDH2-lentiviral vector (*n* = 5 rats per group) were injected during the first day of deprivation **(B)**. The -/ /- symbol in the *x-axis* represents the 15-day deprivation period. The first, second and third re-exposure consumptions were *symbol, means significant different from control-Lenti *p* < 0.001; and ^†^symbol, means significant different from its own baseline value *p* < 0.01 (Panel** A** was adapted from Tampier et al., 2013 and Panel** B**, was adapted from Karahanian et al., 2015).

In the ADE model in UChB rats, chronic ethanol intake for 1–3 months is interrupted by an alcohol deprivation of 7–15 days before animals are allowed ethanol re-access. Upon ethanol re-access, animals consume intoxicating amounts of ethanol of the order of 2–2.5 g ethanol/kg/in the first 60 min of re-access (Tampier et al., 2013; Karahanian et al., 2015). In the UChB model, ethanol intake studies in the ADE condition were aimed at dissociating ADE ethanol intake from the ethanol “drinking-in-the-dark” condition, where high ethanol intakes are observed primarily if water is not offered (Thiele et al., 2014). Since, as shown in Wistar and P rats, the endogenous opiate tone is increased upon food intake (Jalowiec et al., 1981) which is *per se* involved in ethanol intake (Froehlich et al., 1990), the studies in UChB rats were conducted at 1–2 PM (on a 7 AM to 7 PM normal light cycle). Animals rapidly and almost exclusively approach the alcohol solution bottles and not the water bottles, consuming minimal amounts of water upon re-access. A large ADE-induced intake is observed mainly during the initial hour of ethanol re-access (Tampier et al., 2013; Karahanian et al., 2015), in line with studies of Hölter and Spanagel (1999) and Rodd et al. (2009) for HAD and Wistar rats, respectively. The latter authors have demonstrated that on ethanol re-access in the ADE condition, animals are willing to work for alcohol to a greater extent (e.g., to a higher bar-pressing breakpoint), suggesting a more rewarding effect of ethanol in such condition (Hölter et al., 1998). An additional characteristic of the ADE mechanism is “kindling-like” effect that increases the post ADE ethanol intake following several periods of ethanol deprivation and ethanol reinstatement (Hölter and Spanagel, 1999; Rodd et al., 2009), a characteristic also observed in UChB rats (Tampier et al., 2013; Karahanian et al., 2015; Figures 10A,B). Figures 10A,B further show that the deprivation period partly allows the recovery of the inhibitory effect on ethanol intake exerted by the intra-VTA administration of a lentiviral vector coding for an anticatalase shRNA or an ALDH enzyme (Tampier et al., 2013; Karahanian et al., 2015). These findings are in line with the observations of Muggironi et al. (2013) and Orrico et al. (2013) who showed that administration of penicillamine, an acetaldehyde trapping agent, partly inhibited the relapse-like alcohol intake in Wistar rats. Vengeliene et al. (2005) showed in Wistar rats that the i.p. administration of ethanol prior to oral re-access (which *per se* generates acetaldehyde before oral ethanol intake occurs) partly reduced ADE ethanol intake upon re-access.

Overall, studies show that in animals that have consumed ethanol chronically and are subjected to a protracted abstinence followed by ethanol re-access, brain-derived acetaldehyde plays a significant role in the relapse-like drinking. Noteworthy, relapse drinking is a characteristic of alcoholism in humans.

### Maintenance and Relapse-Like Alcohol Drinking: Stem Cells Administration

As indicated above, neuroinflammation leads to cognitive dysfunction and increases chronic alcohol intake (Blednov et al., 2011; Crews and Vetreno, 2016; Montesinos et al., 2016). These studies suggest that reducing neuro-inflammation could reduce both chronic ethanol intake (*maintenance*) and possibly relapse-like drinking. Developments in the stem cell field have shown that most tissues contain mesenchymal stem cells (MSCs; Prockop et al., 2010), known to be activated by inflammatory mediators (e.g., TNFα) in damaged areas, leading to the generation of anti-inflammatory cytokines including IL-10 (Lee et al., 2016) and a soluble TNFα receptor, which neutralizes TNFα (Yagi et al., 2010). MSCs can be isolated and expanded from a number of tissues, such as bone marrow and adipose tissue (Contador et al., 2015; Ezquer et al., 2016).

Yang et al. (2015) showed that hippocampal apoptosis and neurocognitive impairments generated by chronic ethanol administration in Sprague-Dawley rats could be reversed by the infusion of mesenchymal bone marrow stem cells. The study indicated that increases in hippocampal superoxide dismutase (which lowers oxidative stress and likely neuroinflammation) as well as increases in neural growth factor were associated with the reversal of apoptosis and cognitive deficits.

**Figure 11:**
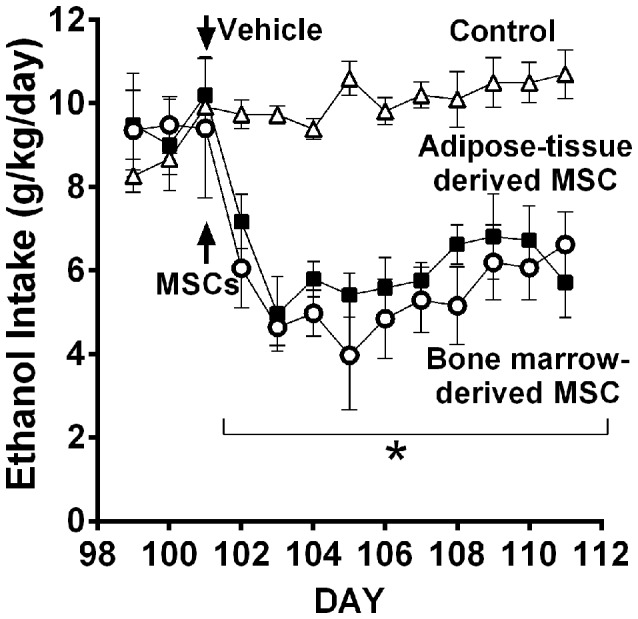
**Intra cerebroventricular injection of bone marrow-derived mesenchymal stem cell (MSC) or adipose tissue—MSC reduces chronic 24-h ethanol consumption in UChB rats**. Rats under chronic ethanol access were injected with bone marrow-derived or adipose tissue—derived MSCs or vehicle into the left cerebral ventricle (*n* = 5 rats per group). Ethanol consumption is shown as g ethanol/kg/day. Asterisk *p < 0.001 indicates a reduction of ethanol intake compared to vehicle during all MSCs post-treatment days (Data from Israel et al., 2017).

Recent work in UChB rats (Israel et al., 2017) tested whether MSCs from bone marrow or adipose tissue of ethanol-naïve rats injected intra-cerebroventricularly could inhibit chronic ethanol intake both in the *maintenance* condition and in the *relapse-like* condition induced by the ethanol deprivation effect (ADE). Figure 11 shows the inhibitory effect of MSCs on intake of ethanol of rats that had freely ingested 10% ethanol for 3 months. Data show that a significant inhibition of ethanol intake exerted by the MSCs; a single dose of MSCs (5 × 10^5^ cells in 5 μL) injected into the brain lateral ventricle reduced by 40%–60% the *maintenance* of chronic alcohol intake for the 10 days studied.

**Figure 12:**
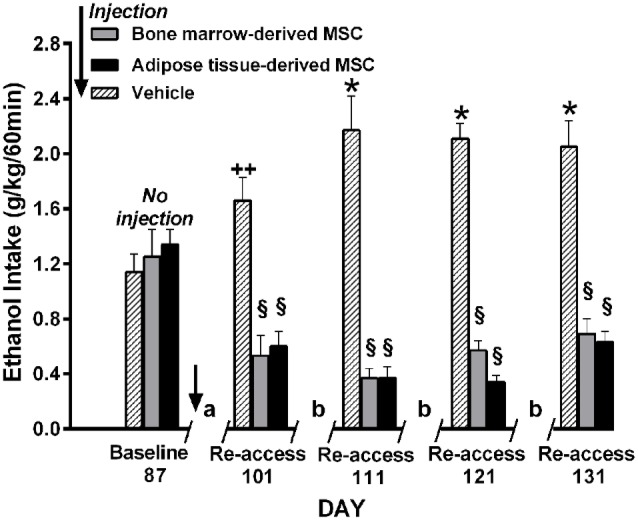
**Intra cerebroventricular injection of bone marrow-derived or adipose-tissue derived MSCs block relapse-like (60-min) ethanol intake in UChB rats**. Rats allowed 87 days of free-choice ethanol access were injected on the fourth day of deprivation with bone marrow-derived MSC (gray columns), adipose tissue-derived MSC (black columns) or vehicle (dashed columns) in the left cerebral ventricle (*n* = 5 rats per group) and deprived of ethanol for 14-days, after which ethanol re-access was allowed. Animals were further subjected to three additional cycles of 3 days of *ad libitum* ethanol drinking and further 7-days of deprivation prior to the next ethanol re-access. The symbol / / represents the deprivation period prior to ethanol re-access: **(a)** 14-day deprivation, **(b)** 7-day deprivation. *p < 0.001 and ^++^*p* < 0.05 indicate ethanol intake increases vs. baseline. ^§^*p* < 0.001 indicates a reduction of ethanol intake compared to baseline value for each of the MSC types (Data from Israel et al., 2017).

*Relapse-like alcohol intake* as affected by MSCs under the ADE condition, was studied in a separate group of UChB rats. Animals that had freely consumed ethanol solutions for 87 days were deprived of ethanol for 14 days. On the fourth day of deprivation animals were administered the MSCs and on deprivation day 15 animals had re-access to ethanol solutions. Prior to the alcohol deprivation, animals displayed a basal alcohol intake of 1.1 g ethanol/kg/60 min (Figure 12), intake which was doubled after repeated alcohol deprivation (ADE) and re-access cycles, reaching 2.2 g alcohol/kg/60 min (equivalent to the consumption of over 10 standard drinks/70 kg in a 1-h sitting). Data in Figure 12 show that animals treated with MSCs reduced up to 80%–85% their relapse-like alcohol intake compared to sham control rats. There were no significant differences between the effects of bone marrow-derived MSCs and adipose tissue-derived MSCs. It is noted that a single intra-cerebroventricular injection of both MSC types inhibited relapse-like drinking for the 40 days investigated, suggesting a marked inhibition of the ADE-activated brain reward systems.

Overall, the inhibition of ethanol intake by MSCs, both under chronic and relapse-like conditions, further supports the view that chronic ethanol intake is maintained by brain oxidative stress/neuroinflammatory conditions, also indicating a role of inflammatory mechanisms on relapse-like ethanol intake. Noteworthy is the long-lasting inhibition afforded by a single administration of MSCs on the relapse-like ethanol intake condition.

## Conclusions

The studies presented:

(a) Confirm, by the use of genetic modifications, studies by several groups in several rat strains that had indicated that catalase-mediated brain oxidation of ethanol into acetaldehyde is required for animals to initiate (*acquisition*) chronic ethanol intake. In the UChB rat, brain-derived acetaldehyde was shown to be an absolute requirement (80%–95%) for the initiation of chronic ethanol intake.
(b) Demonstrate that after a steady chronic ethanol intake (*maintenance*) has been attained, brain acetaldehyde generation is no longer required to perpetuate its intake. This effect seen in UChB and Wistar rats has also been observed in operant ethanol self-administration studies in mice. Following a protracted ethanol deprivation, acetaldehyde is again required to induce a relapse-like condition.
(c) Demonstrate that the daily administration of the antioxidant drug N-acetyl cysteine to UChB rats that have consumed ethanol chronically markedly inhibits (70%–75%) voluntary ethanol intake. Noteworthy, N-acetyl cysteine did not inhibit the initial acquisition of ethanol intake of naïve animals.
(d) Demonstrate in UChB rats that the intracerebral administration of salsolinol—the condensation product of dopamine and acetaldehyde—results in enhanced ethanol reinforcement, leading to binge-like ethanol intakes (up to 3 g ethanol/kg in 60 min, equivalent to 15 drinks/70 kg) and in an enhancement of ethanol motivational effects as shown by the place preference technique.
(e) Demonstrate that a single intra-cerebroventricular administration to UChB rats of mesenchymal stem cells, known to have marked anti-inflammatory and antioxidant properties, inhibited *relapse-like ethanol drinking* by 60%–85% for 40 days.
(f) Overall, studies indicate that ethanol-derived metabolites are by themselves involved in the *acquisition* of ethanol intake; while these metabolites are indirectly involved in *maintenanc*e of chronic ethanol intake *and in relapse-like ethanol drinking*. A new element playing a role in *maintenance and relapse-like* drinking is neuroinflammation, partly mediated by acetaldehyde increasing the diffusion of gut lipopolysaccharide into the systemic circulation and possibly by oxygen radicals generated in the oxidation of salsolinol.

A final note, while many of the studies discussed have been conducted in rodents of different species and strains or in cells, extrapolation of these findings to humans requires caution.

## Author Contributions

All the authors contributed to the writing and approved the final manuscript.

## Conflict of Interest Statement

The authors declare that the research was conducted in the absence of any commercial or financial relationships that could be construed as a potential conflict of interest.
